# Evaluation of 2 culture-based rapid tests used by veterinary practices as a tool for selective treatment of clinical mastitis

**DOI:** 10.3168/jdsc.2026-1011

**Published:** 2026-06-11

**Authors:** Lien Creytens, Sofie Piepers, Sarne De Vliegher

**Affiliations:** M-team and Mastitis and Milk Quality Research Unit, Department of Internal Medicine, Reproduction, and Population Medicine, Faculty of Veterinary Medicine, Ghent University, 9820 Merelbeke, Belgium

## Abstract

Antimicrobial resistance necessitates more judicious antimicrobial use, including in dairy production where mastitis remains the primary driver of antibiotic consumption. Selective treatment of nonsevere clinical mastitis (STCM) requires rapid differentiation between gram-positive and gram-negative pathogens, as only gram-positive infections generally benefit from antimicrobial therapy. Because conventional laboratory culture is too slow for STCM, rapid testing systems for pathogen detection in milk are used on farm (performed and analyzed by farm personnel) or on practice (performed and analyzed at the veterinary practice). This study evaluated the diagnostic performance of 2 culture-based rapid tests (MicroMast and VetoRapid) via an on-practice culture approach as part of a clinical trial, with standard laboratory bacteriology as the gold standard. The performance to distinguish (1) gram-positive from gram-negative bacteria and (2) further pathogen identification was assessed by determining sensitivity, specificity, positive predictive values, negative predictive values, accuracy, and Cohen's kappa values. Both tests (VetoRapid more than MicroMast) reliably distinguished gram-positive from gram-negative pathogens, supporting their use in STCM decision-making. However, their ability to identify specific pathogens was limited, restricting their suitability for broader udder health management decisions.

## Highlights

• Rapid testing is used for selective treatment of nonsevere clinical mastitis.
• It can be applied via an “on-farm culturing” or “on-practice culturing” approach.
• VetoRapid and MicroMast were evaluated as part of on-practice culturing.
• Both tests are reliable for selective treatment (VetoRapid more so than MicroMast).
• Both tests are less suitable for udder health management decisions.

## Graphical Abstract

**Figure:**
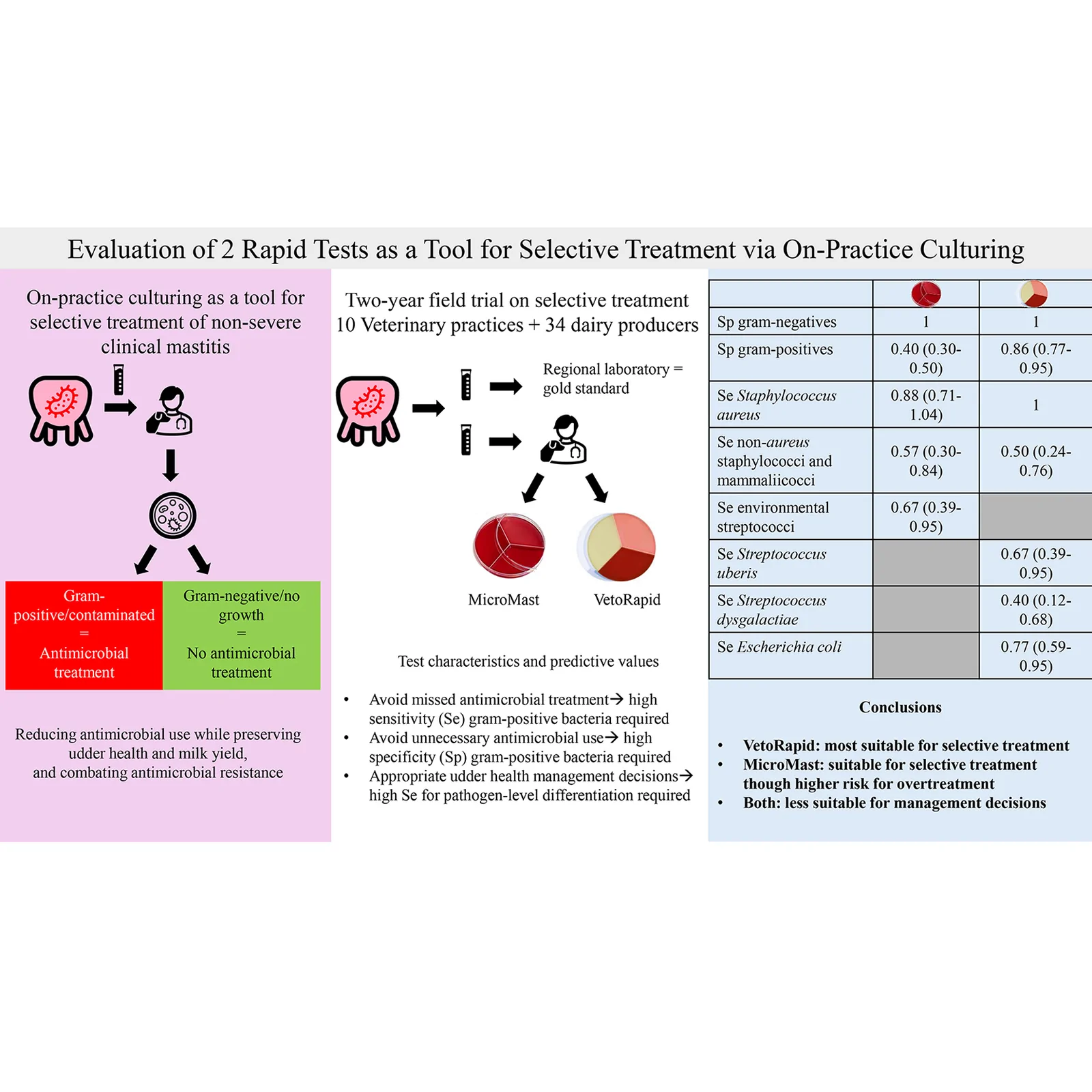
**Summary:** Antimicrobial resistance necessitates more judicious antimicrobial use, including in dairy production, where mastitis remains the primary driver of antibiotic consumption. Selective treatment of nonsevere clinical mastitis requires rapid differentiation between gram-positive and gram-negative pathogens, as only grampositive infections generally benefit from antimicrobial therapy. Conventional laboratory culture is too slow for selective treatment, prompting the development of rapid testing systems for on-farm or on-practice use. This study evaluated the diagnostic performance of 2 culture-based rapid tests (MicroMast and VetoRapid) in the hands of veterinary practices, with standard laboratory bacteriology as the gold standard. Both tests (VetoRapid more so than MicroMast) reliably distinguished gram-positive from gram-negative pathogens, supporting their use in selective treatment decision-making. However, their ability to identify specific pathogens was limited, restricting their suitability for broader udder health management.

Antimicrobial resistance is a threat to public and animal health. In the dairy sector, selective treatment of nonsevere clinical mastitis (**STCM**) has been proposed as an alternative treatment method, as not all cases of clinical mastitis (**CM**) benefit from antimicrobial treatment (**AMT**). In nonsevere cases (e.g., mild [abnormal milk appearance; e.g., flakes, clots, watery] or moderate [abnormal udder appearance; e.g., hard, swollen, red, warm, painful] CM without systemic symptoms [e.g., fever, anorexia, dehydration]), AMT is recommended exclusively when caused by gram-positive (**Gr+**) bacteria. Cases caused by gram-negative (**Gr−**) bacteria (e.g., *Escherichia coli*) should not receive AMT as they are typically resolved by the cows' immune system (Burvenich et al., 2003; Ruegg, 2018). This treatment approach is successful in terms of decreasing antimicrobial usage (**AMU**) without negative impact on udder health and milk production when used on farm (de Jong et al., 2023).

A crucial step in STCM is the reliable differentiation between Gr+, Gr−, and no growth outcomes within 24 h to decide whether AMT is recommended. Standard bacteriological culturing in a regional laboratory can take up to 5 d before results are available, which is too late in terms of STCM. To fill in this gap, a multitude of rapid testing systems (**RTS**) to be used on farm (“on-farm culturing”) or at the veterinary practice (“on-practice culturing” [**OPC**]) have been developed.

MicroMast (**MM**; MicroMast, Světlá nad Sázavou, Tsjech Republic) consists of a nonselective blood agar, a Gr+-specific agar (containing nalidixic acid and colistin), and a MacConkey agar specifically for Gr− bacteria. The nonselective agar enables the growth of aerobic bacteria in addition to yeast, fungi, and *Bacillus* spp. Pathogen identification is possible by growth on a specific sector (Gr+-specific agar or MacConkey agar for Gr− bacteria), the minimum growth time (>24 h for *Corynebacterium* spp. and *Trueperella pyogenes*), and the presence or absence of hemolysis patterns. On the Gr+-specific agar, a catalase test can be used to first distinguish staphylococci (catalase-positive) from streptococci (catalase-negative). Subsequently, an agglutination test can be used to distinguish *Staphylococcus aureus* from non-*aureus* staphylococci and mammaliicocci (**NASM**). In this study, DrySpot Staphytect Plus Latex Agglutination Test (Oxoïd, Aalst, Belgium) was used for this purpose. Differentiation within the *Streptococcus* genus is confined to identifying *Streptococcus agalactiae* separately from the so-called “environmental streptococci,” primarily *Streptococcus uberis* and *Streptococcus dysgalactiae*, based on differences in hemolysis patterns.

VetoRapid (**VR**; Vetoquinol, Lure Cedex, France) consists of 3 selective agar sectors. Sector 1 is a chromogenic agar for Gr− bacteria, containing bile salts and vancomycin to inhibit Gr+ growth. Sector 2 is a modified mannitol salt agar selective for staphylococci allowing the differentiation between *S. aureus* and the most common NASM. Similar to the approach used for MM, an agglutination test (DrySpot Staphytect Plus Latex Agglutination Test, Oxoïd, Aalst, Belgium) was included for VR to verify the distinction between *S. aureus* and NASM (De Visscher et al., 2013). The agglutination test was used for confirmation, particularly when mannitol fermentation was observed. Sector 3 is an agar specific for *Streptococcus* spp. and *Streptococcus*-like organisms (e.g., enterococci), containing modified Edwards agar with crystal violet and polymyxin B, inhibiting the growth of staphylococci and Gr− bacteria. Differentiation between the different streptococci and *Streptococcu*s-like organisms relies on hemolysis patterns and esculin hydrolysis. For both RT, results are classified as contaminated when 3 or more phenotypically distinct bacterial species are observed, which is more readily detected on MM due to its nonselective agar. In the absence of visible bacterial colonies, results are classified as “no growth.” Test characteristics and predictive values for VR have been determined when used by laboratory technicians (Viora et al., 2014) but not for MM, and test characteristics and predictive values when used in veterinary practices are unavailable for both.

The aim of this study was to determine the test characteristics and predictive values of the VR and MM using OPC with standard laboratory bacteriological culturing as the gold standard (**GS**). First, the assessment was made for the differentiation between Gr+ and Gr− bacteria. Second, the rapid test's capacity for further pathogen differentiation was scrutinized.

The data were collected during a 2-yr field trial implementing STCM in the Flemish region of Belgium. Ten veterinary practices were recruited on a voluntary basis through announcements via social media, digital newsletters, and veterinary professional journals. Each veterinary practice recruited dairy producers from their clientele (34 in total), who participated in the local DHI recording system and were willing to implement STCM. The 34 dairy producers were trained by the project staff in recognizing nonsevere CM cases and in collecting aseptic quarter milk samples for the field trial between August 2022 and August 2024.

At the onset of the milking process, duplicate milk samples were taken in sterile vials from each case of mild or moderate CM. Samples were immediately stored in the fridge (3°C–5°C) pending transportation for OPC within 24 h. One milk sample was processed for OPC using one of the 2 rapid tests (**RT**; either VR or MM, randomly allocated across the 10 veterinary practices [each 5 per RT]). Consequently, each practice performed only one of the 2 RT throughout the study. The second sample was shipped by the veterinary practice to the regional laboratory (Milk Control Centre Flanders [**MCC**], Lier, Belgium) for standard bacteriological culturing (GS; as described by Piepers et al., 2007, and Lipkens et al., 2019). Samples for MCC were kept in the freezer (−18°C) until transportation if pickup at the veterinary practice >24 h after sampling was expected.

Veterinarians and their personnel were trained in inoculating milk samples on one of the RT and in analyzing and documenting the results with the assistance of specific laboratory software (MEX@LAB [MEXcellence BV, Sint-Denijs-Westrem, Belgium]). Ten microliters of milk was inoculated on each of the 3 sectors of the RT, followed by an incubation time of 24 h at 37°C under aerobic conditions. When no growth was visible after 24 h, the RT were placed in the incubator for another 24 h and analyzed again at 48 h. There were no restrictions on the brand of incubator used.

Data were stored and calculated using Microsoft Excel version 2412, 2025 (Microsoft Corporation, Redmond, WA). Contaminated results obtained by the RT or GS were excluded from the comparative analysis. Although contamination is reported as a categorical outcome by both methods (i.e., RT and GS), reliable comparison at the pathogen level is not feasible in these cases due to the presence of multiple noninterpretable isolates. In contrast, mixed cultures (visible growth of 2 different bacteria) were retained in the analysis.

Two analytical approaches were applied, resulting in 2 datasets (DS1 and DS2). Importantly, both datasets contained all culture results; however, the definition of a negative RT result differed between them. In dataset 1 (**DS1**), a RT result was classified as negative only when no bacterial growth was observed. If the RT identified any bacterial growth (regardless of whether it matched the GS result), the result was not considered negative. This dataset was constructed to specifically assess the diagnostic performance of the RT in detecting the presence versus absence of bacterial growth. In dataset 2 (**DS2**), a RT result was classified as negative when either (1) no growth was detected or (2) the pathogen (or pathogen group) identified by the RT did not correspond to the GS result. In this approach, a discordant pathogen identification was treated as a false negative for the pathogen detected by the GS. This dataset was constructed to assess agreement at the pathogen (group) level rather than merely the detection of growth. The results of the RT (first, gram differentiation and, second, pathogen identification to some extent) were compared with the GS using DS1 and DS2, separately. Cohen's kappa value, test characteristics [sensitivity (**Se**) and specificity (**Sp**)], predictive values (positive predictive value and negative predictive value), and accuracy with 95% CI were calculated using proportion ±1.96 × √{[proportion × (1 − proportion)]/sample size}. The classification previously proposed by Royster et al. (2014) was used with Se, Sp, and predictive values as low (<0.60), intermediate (0.60–0.80), or high (>0.80). Cohen's kappa value (κ), which is typically used to assess the agreement between 2 raters, was applied to quantify the agreement between the RT and the GS beyond chance and interpreted as proposed by Dohoo et al. (2009).

A total of 206 nonsevere CM cases were sampled (Table 1). Thirteen and 4 samples were contaminated on standard bacteriological culturing and the RT (3 on VR and 1 on MM), respectively, and were excluded from further analysis. This resulted in 189 analyzable RT versus GS sample pairs (110 with MM and 79 with VR) for further evaluation.

**Table 1:** Bacterial causes of nonsevere clinical mastitis cases cultured with standard laboratory bacteriological culture (n = 206) and 2 rapid tests (VetoRapid [n = 90] and MicroMast [n = 116])

Bacteria	Number on standard laboratory bacteriological culture (%)	Number on VetoRapid (%)	Number on MicroMast (%)
Gram-positive	132 (64.1)	49 (54.4)	80 (67)
*Staphylococcus aureus*	30 (14.6)	1 (1.1)	17 (14.6)
Non-*aureus* staphylococci and mammaliicocci	27 (13.1)	12 (13.3)	11 (9.5)
Environmental streptococci	—	21 (23.3)^1^	20 (17.2)^1^
*Streptococcus uberis*	34 (16.5)	4 (4.4)^2^	—
*Streptococcus dysgalactiae*	22 (10.7)	4 (4.4)^2^	—
Enterococci	11 (5.3)	4 (4.4)^2^	—
Aerococci	5 (2.4)	—	—
*Corynebacterium* spp.	2 (0.9)	—	5 (4.3)^3^
*Bacillus* spp.	10 (4.8)	—	2 (1.7)^3^
*Trueperella pyogenes*	3 (1.5)	—	2 (1.7)^3^
Gram-negative	39 (18.9)	14 (15.6)	18 (15.5)
*Escherichia coli*	29 (14.1)	10 (11.1)	—
*Klebsiella pneumoniae*	2 (0.9)	2 (2.2)^4^	—
*Serratia* spp.	2 (0.9)	0 (0)	0 (0)
*Pseudomonas* spp.	2 (0.9)	0 (0)	0 (0)
*Citrobacter* spp.	2 (0.9)	0 (0)	0 (0)
*Enterobacter* spp.	1 (0.5)	0 (0)	0 (0)
Yeasts	6 (3)	0 (0)	0 (0)
Fungi	0 (0)	0 (0)	4 (3.4)
Contaminated	13 (6.3)	3 (3.3)	1 (1)
Culture-negative	22 (10.7)	24 (26.7)	22 (19)

1 Cases where further pathogen identification was not possible (MicroMast) or uncertain (VetoRapid).

2 Only detectable with VetoRapid.

3 Only detectable with MicroMast.

4 Only detectable with VetoRapid as *Klebsiella* spp.

With MM, 73 samples (66.4%) yielded Gr+ bacteria, 16 (14.5%) yielded Gr− bacteria and 21 showed no growth (19.1%). Of the Gr+ results, more *S. aureus* (20; 18.2%) than NASM (14; 12.7%) were detected, next to 32 (29.1%) environmental streptococci. With VR, 45 samples (57%) yielded Gr+ bacteria, 14 (17.7%) yielded Gr− bacteria, and 20 (25.3%) showed no growth. Among the Gr+, more NASM (13; 16.5%) than *S. aureus* (10; 12.7%) were detected, next to 17 (21.5%) *S. uberis* and 7 (8.9%) S. *dysgalactiae.* Among the Gr−, 10 (12.7%) showed growth of *E. coli* (Table 2, Table 3).

**Table 2:** Test characteristics and predictive values of MicroMast and VetoRapid, 2 commercial culture-based rapid tests for pathogen detection in milk, for the detection of gram-positive and gram-negative bacteria compared with standard laboratory bacteriological culture as the gold standard

Item	Gram-positive		Gram-negative	
	MicroMast	VetoRapid	MicroMast	VetoRapid
Dataset 1^1^				
Sensitivity (95% CI)	0.81	0.80	0.93	0.72
	(0.72–0.89)	(0.70–0.90)	(0.83–1.03)	(0.54–0.90)
Specificity (95% CI)	0.40	0.86	1.00	1.00
	(0.30–0.50)	(0.77–0.95)		
Positive predictive value	0.87	0.98	1.00	1.00
	(0.79–0.94)	(0.94–1.02)		
Negative predictive value	0.30	0.38	0.86	0.55
	(0.20–0.40)	(0.25–0.50)	(0.72–0.99)	(0.35–0.74)
Accuracy	0.74	0.81	0.80	0.79
	(0.64–0.83)	(0.70–0.91)	(0.66–0.94)	(0.63–0.95)
Cohen's kappa value	0.18	0.42	0.44	0.57
	(0.10–0.26)	(0.29–0.55)	(0.28–0.60)	(0.37–0.76)
Dataset 2^2^				
Sensitivity (95% CI)	0.77	0.75	0.76	0.59
	(0.70–0.85)	(0.66–0.85)	(0.69–0.84)	(0.48–0.70)
Specificity (95% CI)	0.49	0.81	0.96	0.98
	(0.39–0.58)	(0.72–0.89)	(0.92–0.99)	(0.95–1.01)
Positive predictive value	0.76	0.89	0.76	0.93
	(0.68–0.84)	(0.82–0.96)	(0.69–0.84)	(0.87–0.99)
Negative predictive value	0.50	0.62	0.96	0.86
	(0.41–0.59)	(0.51–0.72)	(0.92–0.99)	(0.79–0.94)
Accuracy	0.68	0.77	0.93	0.87
	(0.59–0.77)	(0.68–0.86)	(0.88–0.98)	(0.80–0.95)
Cohen's kappa value	0.26	0.52	0.72	0.65
	(0.18–0.34)	(0.41–0.63)	(0.64–0.81)	(0.54–0.75)

1 Database using the definition that no growth on the rapid test was considered a negative result.

2 Database using the definition that no growth or growth of a different pathogen group on the rapid test than in the gold standard was considered a negative result.

**Table 3:** Test characteristics and predictive values of MicroMast and VetoRapid, 2 commercial culture-based rapid tests for pathogen detection in milk, for the detection of common mastitis pathogens compared with standard laboratory bacteriological culture

Item	Total staphylococci		*Staphylococcus aureus*		Non-*aureus* staphylococci and mammaliicocci		Total streptococci		Environmental streptococci^1^		*Streptococcus uberis*^2^		*Streptococcus dysgalactiae*^2^		*Escherichia coli*^2^	
	MM^3^	VR^4^	MM^3^	VR^4^	MM^3^	VR^4^	MM^3^	VR^4^	MM^3^	VR^4^	MM^3^	VR^4^	MM^3^	VR^4^	MM^3^	VR^4^
Dataset 1^5^																
Sensitivity (95% CI)	0.82	0.73	0.88	1.00	0.57	0.50	0.84	0.81	0.67	—	—	0.67	—	0.40	—	0.77
	(0.68–0.96)	(0.52–0.94)	(0.71–1.04)		(0.30–0.84)	(0.24–0.76)	(0.72–0.96)	(0.66–0.95)	(0.46–0.87)			(0.39–0.95)		(0.12–0.68)		(0.59–0.95)
Specificity (95% CI)	0.75	1.00	0.75	1.00	1.00	1.00	0.60	0.86	0.75	—	—	1.00	—	1.00	—	1.00
	(0.60–0.90)		(0.54–0.96)				(0.44–0.76)	(0.73–0.99)	(0.56–0.94)							
Positive predictive value	0.90	1.00	0.78	1.00	1.00	1.00	0.84	0.94	0.80	—	—	1.00	—	1.00	—	1.00
	(0.79–1.01)		(0.57–0.98)				(0.72–0.96)	(0.86–1.03)	(0.62–0.98)							
Negative predictive value	0.60	0.60	0.86	1.00	0.67	0.60	0.60	0.60	0.60	—	—	0.86	—	0.67	—	0.67
	(0.42–0.78)	(0.37–0.83)	(0.69–1.03)		(0.41–0.92)	(0.34–0.86)	(0.44–0.76)	(0.42–0.78)	(0.39–0.81)			(0.65–1.06)		(0.40–0.93)		(0.47–0.87)
Accuracy	0.80	0.81	0.81	1.00	0.77	0.71	0.77	0.82	0.70	—	—	0.89	—	0.73	—	0.84
	(0.66–0.94)	(0.62–1)	(0.62–1)		(0.54–1)	(0.48–0.95)	(0.63–0.91)	(0.68–0.96)	(0.50–0.90)			(0.70–1.07)		(0.48–0.98)		(0.69–1)
Cohen's kappa value	0.53	0.61	0.63	1.00	0.55	0.46	0.44	0.58	0.40	—	—	0.73	—	0.42	—	0.68
	(0.35–0.70)	(0.40–0.82)	(0.39–0.86)		(0.28–0.82)	(0.20–0.72)	(0.28–0.60)	(0.40–0.77)	(0.19–0.61)			(0.44–1.02)		(0.13–0.71)		(0.47–0.89)
Dataset 2^6^																
Sensitivity (95% CI)	0.55	0.52	0.37	0.10	0.29	0.36	0.66	0.74	0.25	—	—	0.12	—	0.33	—	0.67
	(0.45–0.64)	(0.42–0.63)	(0.26–0.47)	(0.03–0.17)	(0.20–0.37)	(0.26–0.47)	(0.57–0.75)	(0.64–0.84)	(0.17–0.33)			(0.05–0.19)		(0.23–0.44)		(0.56–0.77)
Specificity (95% CI)	0.88	0.98	0.90	1.00	0.93	0.90	0.79	0.72	0.87	—	—	0.98	—	0.97	—	1.00
	(0.82–0.94)	(0.96–1.01)	(0.84–0.97)		(0.88–0.98)	(0.83–0.96)	(0.72–0.87)	(0.62–0.82)	(0.81–0.93)			(0.96–1.01)		(0.94–1.01)		
Positive predictive value	0.67	0.92	0.44	1.00	0.36	0.36	0.57	0.50	0.44	—	—	0.67	—	0.50	—	1.00
	(0.58–0.75)	(0.86–0.97)	(0.33–0.55)		(0.27–0.45)	(0.26–0.47)	(0.47–0.66)	(0.39–0.61)	(0.35–0.54)			(0.56–0.77)		(0.39–0.61)		
Negative predictive value	0.82	0.85	0.87	0.88	0.90	0.90	0.85	0.88	0.74	—	—	0.80	—	0.95	—	0.93
	(0.75–0.89)	(0.78–0.92)	(0.80–0.95)	(0.81–0.96)	(0.84–0.96)	(0.83–0.96)	(0.78–0.92)	(0.81–0.95)	(0.66–0.82)			(0.71–0.89)		(0.90–1)		(0.87–0.98)
Accuracy	0.78	0.86	0.81	0.89	0.85	0.82	0.75	0.73	0.69	—	—	0.80	—	0.92	—	0.94
	(0.70–0.86)	(0.79–0.93)	(0.62–1)	(0.82–0.96)	(0.78–0.91)	(0.74–0.91)	(0.67–0.83)	(0.63–0.82)	(0.60–0.78)			(0.71–0.89)		(0.87–0.98)		(0.88–0.99)
Cohen's kappa value	0.45	0.59	0.29	0.16	0.23	0.26	0.43	0.40	0.14	—	—	0.14	—	0.36	—	0.76
	(0.36–0.55)	(0.48–0.70)	(0.20–0.37)	(0.08–0.24)	(0.16–0.31)	(0.16–0.36)	(0.34–0.52)	(0.30–0.51)	(0.08–0.20)			(0.07–0.22)		(0.26–0.47)		(0.67–0.86)

1 Only for MicroMast, as *Streptococcus uberis* and *Streptococcus dysgalactiae* can be identified separately with VetoRapid.

2 Only for VetoRapid, as *S. uberis, S. dysgalactiae*, and *Escherichia coli* cannot be separately identified with MicroMast.

3 MicroMast.

4 VetoRapid.

5 Database using the definition that no growth on the rapid test was considered a negative result.

6 Database using the definition that no growth or growth of a different pathogen group on the rapid test than in the gold standard was considered a negative result.

Predictive values are often the most informative metrics to evaluate diagnostic field tests. However, they depend directly on disease prevalence. Given the variable prevalence of nonsevere CM among dairy farms (Verbeke et al., 2014), prevalence-independent test characteristics including Se and Sp were emphasized in this study. In DS1, a negative RT result was defined only as the absence of bacterial growth; discordant pathogen identifications were not considered as false negatives. This reduced false-negative and false-positive observations for several pathogens, particularly in DS1, and may have influenced the precision of some diagnostic performance estimates. No a priori sample size calculation was performed because case inclusion depended on eligible CM cases during the study period, reflecting the field-based study design. Precision of the estimates was therefore assessed post hoc using 95% CI. Wide CI for less frequently isolated pathogens indicate substantial statistical uncertainty, and these results should be interpreted with caution.

To justify the use of a RT within a STCM strategy, high Sp is particularly required for Gr+ pathogens. Specificity for Gr− pathogens remains important but is of lesser relevance, given that culture-negative results lead to the same treatment recommendation. In this study, the Sp for Gr− is high for VR (1 in DS1 and 0.98 in DS2) and MM (1 in DS1 and 0.96 in DS2). The Sp for Gr+ is high for VR (0.86 in DS1 and 0.81 in DS2), yet lower for MM (0.40 in DS1 and 0.49 in DS2), increasing the likelihood of falsely diagnosing a CM case as caused by a Gr+ bacterium with MM, leading to unnecessary AMT. The Sp for specific Gr+ bacteria are generally high for MM as well as for VR. The Sp for MM is low for Gr+, yet acceptable for specific pathogen identification. The reduced Sp for Gr+ bacteria observed with MM may partly be explained by the higher proportion of “unspecified” results and the broader classification used in the aggregated Gr+ analysis. Several false-positive streptococcal results were observed, including environmental streptococci and a substantial number of unspecified outcomes, indicating that borderline or ambiguous classifications contributed to discordant results. Such discrepancies are more likely when the RT and the GS are performed on separate milk samples, particularly in the presence of low bacterial loads or after sample freezing. In addition, the use of a Gr+ selective culture medium in MM may facilitate the detection of low-level Gr+ bacterial growth by suppressing competing flora, whereas nonselective blood agar used in routine laboratory culture may fail to detect these low bacterial loads. Together, these factors may inflate apparent false-positive results when Gr+ pathogens are analyzed as an aggregated group. In contrast, classification at the individual pathogen level (e.g., *S. aureus* or streptococci) is more restrictive, so ambiguous results are less often assigned to a specific pathogen and are more frequently classified as true negatives, resulting in higher Sp estimates.

While high Sp for the detection of Gr+ pathogens is essential within a STCM strategy to avoid unnecessary AMU, adequate Se is equally important. Gram-positive intramammary infections generally require AMT, and failure to identify these pathogens may reduce the likelihood of clinical and bacteriological cure as was recently demonstrated by Krömker et al. (2025). In the present study, both VR and MM Se exceeded 75% for the detection of Gr+ pathogens, indicating that the majority of Gr+ infections were correctly identified by the RT. This level of Se supports their clinical applicability within an STCM framework, as most cows requiring AMT would be appropriately identified, while the risk of undertreatment remains limited.

Beyond supporting treatment decisions, RTS-based pathogen identification could contribute to improving udder health management, for example, in decisions on culling of a specific animal or refinement of herd-specific mastitis prevention strategies. Such broader applications demand a high Se for major mastitis pathogens. Specific pathogen identification with MM is limited to *S. aureus* and NASM. Although the Se for NASM is low in both datasets (0.57 in DS1 and 0.29 in DS2), it varies considerably for *S. aureus* between the 2 datasets (0.88 in DS1 and 0.37 in DS2). This substantial discrepancy indicates *S. aureus* generally exhibits comparable growth on the RT and MCC, yet the RT appears more susceptible to misclassification with other staphylococci. In contrast, VR is capable of differentiating *S. aureus*, NASM, *S. uberis*, and *S. dysgalactiae*, with only acceptable Se for *S. aureus* (in DS1). *Escherichia coli* can only be differentiated with VR, revealing acceptable Se in both datasets. Low Se could result in a high frequency of misdiagnosed or undetected cases, posing risks for critical decisions such as not culling a *S. aureus*-infected cow that was left undetected. Therefore, management decisions based on VR and MM should be approached cautiously, as also substantiated by the low κ-values (Table 3).

Our findings for Se and Sp for Gr+ and Gr− are in the same range of those found in studies evaluating other RTS, except for the Se for Gr− of MM (lower in our study; McDougall et al., 2018; Malcata et al., 2021). Sensitivity and Sp for the pathogen-specific identification with VR align with Viora et al. (2014). Also, other RTS showed comparable Se and Sp for pathogen-specific identification, but higher Se for NASM and *S. uberis* were found with Mastatest (McDougall et al., 2018; Jones et al., 2019; Malcata et al., 2021). However, caution is needed when comparing results of different studies due to variation in study design, RTS, and pathogen distribution.

Distinguishing *S. aureus* from other staphylococci is challenging in the veterinary practice, independent of the RT. Still, validation of the outcome of the GS method using PCR or MALDI-TOF MS was not included in the current work, which is a shortcoming. Because duplicate sampling was used, it is also conceivable that *S. aureus* was isolated in the laboratory from the second sample, whereas the first sample, which was used for the RT, may not have contained *S. aureus* at a concentration that was high enough to allow growth. Also, milk sample storage and transport might have introduced bias. Some, yet not all, milk samples destined for the GS laboratory were frozen before transport, and those for the RT were always fresh. This could have influenced the detection of specific pathogens such as *S. aureus* and *E. coli* (Schukken et al., 1989; Villanueva et al., 1991). As the number of samples that were frozen before transportation was not recorded, the extent of this effect cannot be determined. Additionally, the greater proportion of contaminated milk samples in the standard laboratory cultures relative to the RT results may be attributable, at least in part, to longer transport times for the laboratory samples (Royster et al., 2014).

In this study, selective RTS were used. The main advantage of the VR and MM systems is that they incorporate multiple selective media on a single plate, allowing straightforward visual differentiation between Gr+ and Gr− bacteria without the need for ancillary tests. When only blood agar is used, differentiation between Gr+ and Gr− organisms generally requires an additional step, such as a potassium hydroxide test, or the use of an additional MacConkey plate. Although blood agar itself is typically less expensive than selective media, this approach therefore requires either additional plates or extra tests, which may increase workload and require additional training in OPC settings. Combination plates containing multiple media (e.g., Minnesota Easy Culture System II Bi-Plate and Tri-Plate [University of Minnesota Laboratory for Udder Health, St. Paul, MN]) have been shown to perform well under field conditions (Sipka et al., 2021). However, these systems are currently difficult to obtain in Europe, including Belgium, which limits their practical implementation for OPC.

We conclude that both VR and MM reliably distinguish Gr+ from Gr− bacteria under OPC conditions, and can be used to implement STCM. VetoRapid has a slight advantage due to its higher specificity and lower risk of unnecessary AMU. The limited pathogen-level differentiation in both tests reduces their applicability for udder health-related management decisions.
